# Feasibility of iliosacral screw placement in patients with upper sacral dysplasia

**DOI:** 10.1186/s13018-019-1472-7

**Published:** 2019-12-09

**Authors:** Christoph J. Laux, Lizzy Weigelt, Georg Osterhoff, Ksenija Slankamenac, Clément M. L. Werner

**Affiliations:** 1 Department of Trauma Surgery, University Hospital Zurich, University of Zurich, Rämistrasse 100, 8091 Zurich, Switzerland; 2 Department of Orthopaedics, Balgrist University Hospital, University of Zurich, Forchstrasse 340, 8008 Zurich, Switzerland; 3 Department of Orthopaedic, Trauma and Plastic Surgery, University Hospital Leipzig, Liebigstrasse 20, 04103 Leipzig, Germany; 4 Institute of Emergency Medicine, University Hospital Zurich, University of Zurich, Rämistrasse 100, 8091 Zurich, Switzerland

**Keywords:** Iliosacral screw placement, Pelvic ring injury, Upper sacral dysplasia, Sacral dysmorphism, Radiographic signs, Safety

## Abstract

**Background:**

Exact knowledge of the sacral anatomy is crucial for the percutaneous insertion of iliosacral screws. However, dysplastic anatomical patterns are common. In addition to a preoperative computed tomography (CT) analysis, conventional radiographic measures may help to identify upper sacral dysplasia and to avoid damage to surrounding structures. Aiming to further increase safety in percutaneous iliosacral screw placement in the presence of sacral dysmorphism, this study examined the prevalence of previously established radiographic signs and, in addition, defined the “critical SI angle” as a new radiographic criterion.

**Methods:**

Pelvic CT scans of 98 consecutive trauma patients were analysed. Next to assessment of established signs indicating upper sacral dysplasia, the critical sacroiliac (SI) angle was defined in standardized pelvic outlet views.

**Results:**

The critical SI angle significantly correlates with the presence of mammillary bodies and an intraarticular vacuum phenomenon. With a cut-off value of − 14.2°, the critical SI angle detects the feasibility of a safe iliosacral screw insertion in pelvic outlet views with a sensitivity of 85.9% and a specificity of 85.7%.

**Conclusions:**

The critical SI angle can support the decision-making when planning iliosacral screw fixation. The clinical value of the established signs of upper sacral dysplasia remains uncertain.

## Introduction

Exact knowledge of the sacral anatomy is crucial for the percutaneous insertion of iliosacral screws for fixation of posterior pelvic ring injuries. Despite its widespread use, this technique remains demanding. Due to the long screw trajectory and the proximity of neurovascular structures, even deviations of only a few degrees bear the risk of a cortical breach and neurovascular complications. This is true especially in patients with a dysmorphic upper sacral anatomy [1]. The lumbosacral nerve roots are the structures most at risk [1–3]. Miller and Routt identified radiographic signs indicating upper sacral dysmorphism on pelvic outlet and lateral plain films (collinearity, mammillary processes, noncircular and misshapen anterior first sacral neuroforamina, residual disc space S1/S2) [4]. However, their reliability and clinical value for preoperative planning of iliosacral screw fixation remains unknown. To achieve adequate and safe fixation in dysplastic upper sacral segments, iliosacral screw insertion into the S2 segment was recommended [5–8]. As such dysplastic patterns are very common [9–11], preoperative computed tomography (CT) analysis is regarded to be mandatory in order to identify them and to consequently avoid damage to surrounding structures [6].

Aiming to further increase safety in percutaneous iliosacral screw placement in the presence of sacral dysmorphism, this study examined the prevalence of previously established radiographic signs and, in addition, defined the “critical SI angle” as a new radiographic criterion in this context.

## Material and **m**ethods

### Patients

For this retrospective cohort study, pelvic computed tomography (CT) scans of consecutive trauma patients younger than 50 years of age, who were transferred to our level I trauma centre between January 2010 and December 2012, were analysed. To facilitate our measurements, only those patients without a significant pelvic trauma (Young and Burgess LC II, LC III, APC II, APC III and VS type injuries), without dislocated or intraarticular sacral fractures and without ankylosis of the sacroiliac (SI) joints were included. In patients with metallic implants in the posterior pelvic ring or the lumbosacral junction, the affected side was excluded due to image artefacts. All patients beyond the age of 50 years were excluded to reduce a possible image bias due to degenerative alterations of the pelvic ring such as osteophytic extensions or static compensatory mechanisms that are associated with degenerative disc disease. Ninety-eight patients were included. Three SI joints were excluded because of unilateral fractures with joint involvement. A lumbosacral transitional vertebra impeded the radiological measurements in two SI joints, which were excluded accordingly. Thus, 191 SI joints were considered in the analysis.

### Radiological analysis

**Fig. 1:**
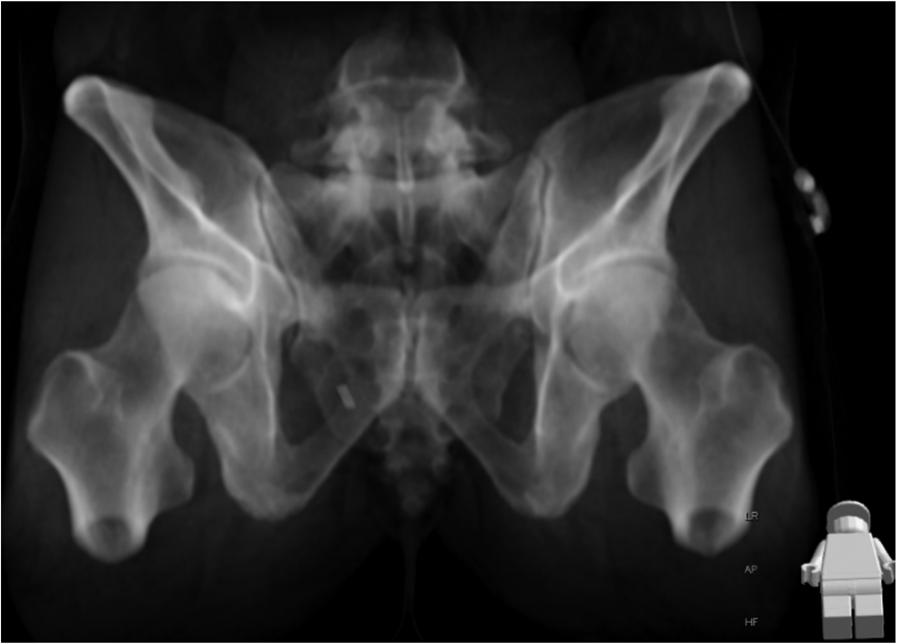
Reconstruction of an outlet ap-view from a full body CT scan using maximum intensity projections (MIP)

**Fig. 2:**
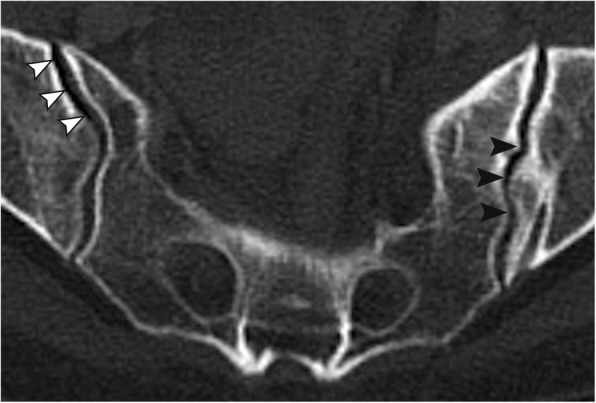
Intraarticular vacuum phenomenon as a sign of joint degeneration (white arrow heads) and a tongue-in-groove pattern (black arrow heads) as a sign for upper sacral dysplasia [4]

In the acute trauma setting, plain radiographs are associated with poor projections and visualization due to insufficient patient positioning and extracorporeal foreign material. In order to obtain comparable views of the pelvis without obstructive extracorporeal items, we acquired standardized outlet views generated from three-dimensional reconstructions of the CT image data using maximum intensity projections (MIP) of the pelvis (Fig. 1). Due to the individual spinopelvic geometry, the sagittal projection angle needed for a “true” pelvic outlet view is individual [12]. Images were aligned accordingly using a multiplanar reformation (MPR) tool to achieve a projection of the symphysis on the median sagittal plain with the upper rim centred on the body of the S2 vertebra [13]. The coronal and axial CT planes were adjusted to the axis of the S1 vertebra to best detect and visualize mammillary bodies and non-spherical neuroforamina. Radiographic signs indicating upper sacral dysmorphism as defined by Miller and Routt as well as the presence of an intraarticular vacuum phenomenon as a possible indicator of joint degeneration were assessed (Fig. 2).

The authors did assess the technical feasibility of an iliosacral S1 screw based on reconstructed outlet views. If a horizontal screw (parallel to the S1 vertebral end plate) with a 7.3-mm diameter aiming at the S1 vertebral body was fully covered by bone, screw placement was rated to be feasible. If the screw was only partially uncovered by bone in a horizontal manner, but sufficient bony coverage within the S1 vertebra could be achieved by using a modified, ascending screw trajectory, screw placement was rated to be limitedly feasible. If, irrespective of the screw angulation, no sufficient bony coverage could be attained, screw placement was judged as impossible.

**Fig. 3:**
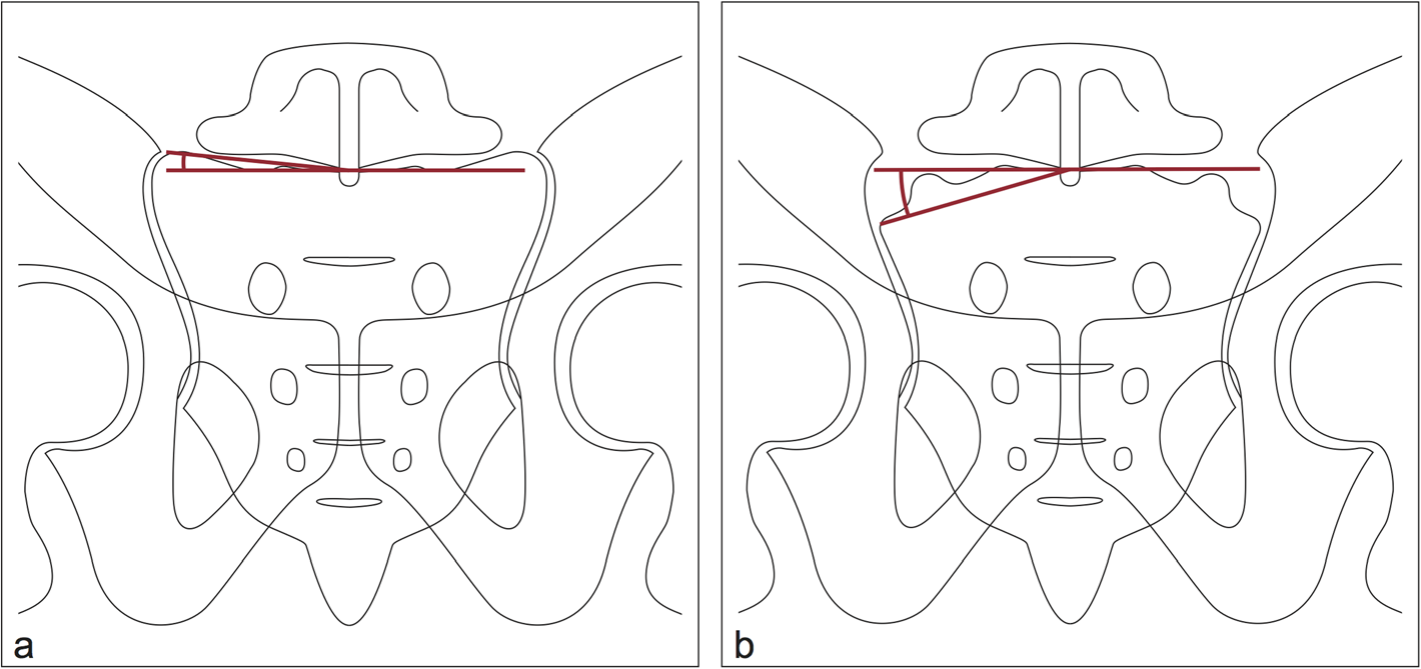
Illustration of the critical SI angle in a normal (**a**) and a dysplastic (**b**) upper sacral segment

**Fig. 4:**
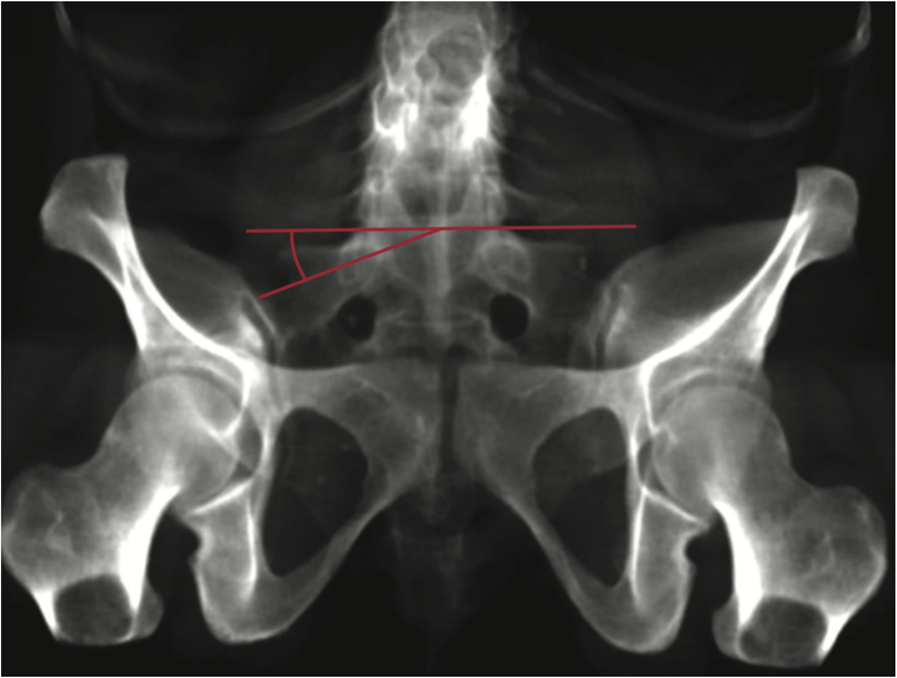
Practical application of the critical SI angle on a reconstructed outlet view with a dysplastic upper sacral segment (CSIA = -20°)

For further anatomic evaluation, the critical sacroiliac (SI) angle was defined (Fig. 3). The first ray of this angle is a horizontal line parallel to the S1 vertebral end plate. The second ray connects the centre of the S1 vertebral end plate with the most lateral portion of the sacral ala before constituting the SI joint. The angle was measured by the first and senior authors CL, LW and CW as exemplified in Fig. 4.

### Statistical analysis

Data was collected in spreadsheets (Microsoft Excel 2010, Microsoft Corporation, Redmond, USA). All statistical analyses were performed using Stata software (version 14, StataCorp. LLC, College Station, Texas). Both the distribution of independent variables and the association of the critical SI angle with the established signs of sacral dysmorphism were assessed with descriptive statistics. We compared all endpoints between groups using simple and multivariable logistic regression models adjusting for potential confounders such as age and sex. In multiple testing, the significance level was corrected according to Bonferroni using a *p* value < 0.017. The cut-off value for the critical SI angle was calculated using the area under the curve (AUC). In addition, the interobserver reliability using Cohen’s kappa [14] and the intraclass correlation coefficient were determined [15].

## Results

The study population showed a median age of 34 (± 11) years and a sex ratio (m:f) of 1.5. The critical SI angle is significantly associated with the presence of mammillary bodies on both sides (Table 1). However, none of the other signs of sacral dysmorphism as described by Routt et al. were found to correlate with it. Furthermore, the critical SI angle was demonstrated to be associated with the presence of an intraarticular vacuum phenomenon in the SI joints (Table 1). A side-specific calculation of the respective association of the critical SI angle (CSIA) to sacral dysmorphic signs did not yield new results of significance (Additional file 1). The interobserver reliability showed a moderate to perfect agreement according to Altmann with Cohen’s kappa being 0.42 (right) and 1.00 (left) [14]. The intraclass correlation coefficient according to Fleiss showed excellent agreement for both sides (0.99 and 0.95, respectively) [15].

**Table 1:** Association of the critical SI angle (CSIA) with signs of upper sacral dysplasia and an intraarticular vacuum phenomenon. Results were adjusted for age and sex as possible confounders. Adjusted differences between sacroiliac joints with and without the respective dysmorphic sign are presented as median with 95% confidence interval

Signs of upper sacral dysplasia			Critical SI angle
Mammillary bodies	No *n* = 152	Yes *n* = 39	−10.4° (−15.6° to −5.2°); ***p*** **< 0.001**
Tongue−in−groove	No *n* = 178	Yes *n* = 13	3.4° (−5.4° to 12.2°); *p* = 0.447
Intervertebral disc	No *n* = 42	Yes *n* = 149	−3.5° (−8.8° to 1.8°); *p* = 0.193
Collinearity	No *n* = 184	Yes *n* = 7	−8.9° (−20.5° to 2.6°); *p* = 0.129
Unspherical neuroforamina	No *n* = 123	Yes *n* = 68	−0.7° (−5.4° to 4.0°); *p* = 0.769
Intraarticular vacuum phenomenon	No *n* = 108	Yes *n* = 71	−6.8° (−11.3° to 2.4°); ***p*** **= 0.003**

Data in bold signifies *p*-value <0.05.

For the preoperative judgement of the feasibility of iliosacral screw placement, the critical SI angle is an auxiliary parameter with a cut-off value of − 14.2° with AUC 0.8722 (95% CI 0.807–0.919) (Table 2). Sensitivity and specificity of the critical SI angle at this cut-off are 85.9 % and 85.7 %, respectively.

**Table 2:** Cut-off of the critical SI angle (CSIA). Results were adjusted for age and sex as possible confounders

	Screw placement impossible *n* = 21	Screw placement feasible *n* = 135	Unadjusted RR (95% CI, *p* value)	Adjusted RR (95% CI, *p* value)
CSIA ≥ −14.2°	3 (14.3%)	116 (85.9%)	36.6 (9.8 to 136.4) *p* < 0.001	37.9 (10.0 to 143.5) *p* < 0.001

*RR* relative risk

Multiple testing showed that the critical SI angle allows for discrimination between impossible and feasible screw placement as well as between feasible and limitedly feasible (using a modified screw angulation) screw placement on both sides (Table 3). However, cases with impossible and limitedly feasible iliosacral screw insertion cannot be discriminated based on the critical SI angle.

**Table 3:** Multiple testing of the critical SI angle (CSIA) when compared to feasibility of iliosacral screw insertion. Significance level (*p* value) has to be corrected according to Bonferroni: significant *p* value < 0.017

	Impossible (group 1)	Feasible (group 2)	Limited feasible (group 3)	Group 1 vs. 2 Adjusted difference (95% CI), *p* value	Group 1 vs. 3 Adjusted difference (95% CI), *p* value	Group 3 vs. 2 Adjusted difference (95% CI), *p* value
CSIA left (°)	*n* = 9 −29.3 (−31.7 to −29.2)	*n* = 69 −2.9 (−10.4 to 9.6)	*n* = 18 −16.3 (−22.4 to −9.4)	23.8 (14.0 to 33.5) *p* < 0.001	8.4 (−2.8 to 1936) *p* = 0.134	13.8 (6.8 to 20.8) *p* < 0.001
CSIA right (°)	*n* = 12 −23.0 (−27.3 to −16.2)	*n* = 66 −2.5 (−10.1 to 10.1)	*n* = 18 −14.6 (−19.6 to −8.1)	19.1 (10.5 to 27.8) *p* < 0.001	4.9 (−4.5 to 14.3) *p* = 0.294	11.3 (3.8 to 18.8) *p* = 0.004

## Discussion

According to Routt, safe iliosacral screw placement relies upon three criteria: accurate reduction of the posterior pelvic ring, thorough understanding of the posterior pelvic anatomy and its variations, and its fluoroscopic visualization of the posterior pelvic ring using standard projections (inlet, outlet, true lateral) [9].

In this study, we found the critical SI angle to facilitate understanding of the posterior pelvic anatomy and to support the decision-making when planning an iliosacral screw fixation in patients with dysplastic upper sacral anatomy. With a sensitivity and specificity of 85.9% and 85.7%, respectively, the critical SI angle predicts the feasibility of safe iliosacral screw insertion in pelvic outlet views. Moreover, based on the critical SI angle, the surgeon can choose to place iliosacral screws using either a traditional or a modified trajectory into S1 or a S2 trajectory instead. As with any radiographic parameter, possible interreader variations need to be taken into account, especially for measurements close to the cut-off value. In our interreader reliability calculation, we found a moderate to perfect agreement. Even at its cut-off value, the precision and the discriminatory power of the critical SI angle was solid and showed a sufficient sensitivity and specificity.

Radiographic tools for the assessment of the feasibility of iliosacral screw insertion have been proposed before. The lateral sacral triangle method as described by Mendel et al. elaborately investigates the presence of safe S1 corridors for the strictly transverse insertion of iliosacral screws [16]. However, the possibility of angulated screw trajectories is not taken into account. The acute alar slope as delineated by Routt et al. is a qualitative criterion and does not provide cut-off values with regard to the feasibility of iliosacral screw placement [9]. However, as stated before, lateral sacral imaging is still critical for intraoperative understanding of the sacral alar anatomy and for facilitation of safe screw insertion.

Because sacral dysplasia typically affects the anterosuperior portion of the sacral ala, the amount of dysplasia might be underestimated on standard ap-radiographs and is best displayed in the outlet projection. Additionally, Kaiser et al. concluded that the coronal and axial angulation of the upper sacral segment accurately discriminates dysmorphic sacra [11]. Accordingly, we chose to reconstruct pelvic outlet views rather than more easily obtainable standard ap-views. Gardner et al. showed in their series of 50 pelvises that the upper sacral safe zone for iliosacral screw insertion is 36% smaller and more oblique in dysplastic sacra [17]. Even though this precluded transverse screw placement in all dysmorphic S1 segments, there was sufficient cross-sectional area within the ala in 91% of patients. As a consequence of the obliquely oriented safe zones, Gardner et al. concluded that the standard fluoroscopic markers of sacral dysmorphism must be interpreted differently when placing iliosacral screws. This corresponds to the present results of our analysis. The screw obliquity required was numbered 30° caudal to cranial on the pelvic outlet view and 15° posterior to anterior on the pelvic inlet view. In cases presenting a critical SI angle below the cut-off value of -14°, a C-clamp might be superior to percutaneous screw fixation as primary surgical stabilization measure.

The suitability of the classical radiographic signs of sacral dysplasia in the assessment of iliosacral screw placement has not been investigated so far. With a prevalence of up to 78% (residual disc space S1/S2) in this series of asymptomatic individuals, the significance of these signs should be scrutinized in accordance to the literature [18]. In this regard, the slight male predominance of this trauma population can probably be neglected.

When defining the critical SI angle, we decided to aim the second ray to the most lateral aspect of sacral ala rather than using a tangent to the ascending (or descending) upper alar margin because the tangent underestimates flat but very medial defects and overestimates steep and very lateral defects. Moreover, the tangent is more sensitive to changes of the projection angle in the outlet view.

The literature on pelvic ring disruptions is based largely on radiographic images [19]. Many authors have suggested radiographic measurements of displacement in pelvic ring disruptions using pelvic outlet radiographs [20, 21]. However, the reliability of these measurements has been found to be moderate. This is a well-known and well-investigated limitation. We therefore aimed to enhance the accuracy of our measurements by using standardized CT reconstructions that are easily obtainable. The critical SI angle has been investigated in standardized “true” pelvic outlet views, which are not standard projections in the acute trauma setting. Then again, since the strong implementation of whole-body computed tomography in the diagnostic workup of polytraumatized patients due to increased survival pelvic CT data is commonly available and can be easily accessed for MIP reconstructions by the trauma radiologist [22, 23].

With increasing displacement, it becomes more and more difficult to ascertain any radiographic parameter in fractured or disrupted pelvises. In case of large displacement (i.e. vertical shear injuries, severely displaced or comminuted sacral fractures), it is therefore suggested to get radiographs in a pelvic binder—with or without traction.

By contrast, intraoperative guidance is usually provided by fluoroscopy [9]. Here, inlet, outlet and true lateral projections are routinely employed during iliosacral screw fixation. Accordingly, pelvic outlet views considering the individual spinopelvic anatomy can also be obtained by preoperative fluoroscopy as an alternative to CT-based reconstructions. Determination of the critical SI angle can then support the screw vectoring.

The most substantial limitation of this study is that the screw placement in S1 is simply assessed empirically. However, this reflects the clinical practice in acute trauma care and more elaborate measurements such as three-dimensional planning of optimal screw positioning often are inappropriate. The critical SI angle offers a quick and easy method to evaluate the sacral anatomy from a surgeon’s point of view. Moreover, in contrast to the so far established signs of sacral dysmorphism, the critical SI angle is an objective and quantifiable parameter. Thus, it may also be used for comparative and scientific purposes.

In our analysis, the critical SI angle is not capable of adequately discriminating cases with impossible and limited feasible screw placement. This information would be desirable considering that 21% of the sacral alae (14% of patients) investigated are judged not being amenable to iliosacral screw fixation. In the analysis by Gardner et al., this group represents 9% of patients. In this regard, our results with use of the critical SI angle are not critically different to the elaborate measurements conducted Gardner and colleagues.

Intraarticular gas, also referred to as vacuum phenomenon, has been associated to degenerative changes before, especially in the elderly [24–27]. The association of intraarticular gas formation in the SI joint and a far negative critical SI angle might represent the joint degeneration due to relative overload in SI joints with sacral alar dysplasia. Even though the clinical relevance is uncertain, this observation might help explain these pathomorphological changes.

In summary, this article suggests a newly defined parameter with the aim to reduce critical complications on the one hand and to advance the anatomical comprehension of this biomechanically important area. However, with this being a first description an analysis of inter- and intrareader validity and reliability is still lacking and provides another scientific incentive.

## Conclusion

The critical SI angle supports the decision-making when planning an iliosacral screw fixation in patients with dysplastic upper sacral anatomy. In this regard, the clinical value of the established signs remains uncertain. When finding a critical SI angle below the cut-off value of -14°, alternative measures for stabilization of the posterior pelvic ring should be considered. In addition, the critical SI angle might predict degenerative changes in the sacroiliac joints.

## Data Availability

All data analysed during this study are included in this published article and its supplements.

## Abbreviations

AUC: Area under the curve CSIA Critical SI angle CT Computed tomography MIP Maximum intensity projections RR Relative risk SI Sacroiliac
